# Current Knowledge on Tick-Borne Encephalitis Virus Interaction with Ticks: Acquisition, Dissemination, and Persistence

**DOI:** 10.3390/pathogens15050535

**Published:** 2026-05-15

**Authors:** Gabrielle Trozzi, Charlotte Sohier, Nick De Regge

**Affiliations:** Exotic and Vector-Borne Diseases, Sciensano, Groeselenberg 99, 1180 Brussels, Belgium; charlotte.sohier@sciensano.be (C.S.); nick.deregge@sciensano.be (N.D.R.)

**Keywords:** tick-borne encephalitis virus, virus–vector interactions, vector competence, virus acquisition, virus dissemination

## Abstract

Tick-borne encephalitis virus (TBEV) is a major arthropod-borne flavivirus responsible for severe neurological disease in humans across Europe and Asia. It is maintained in nature through complex interactions within ticks and between tick vectors, vertebrate hosts and environmental factors. This review summarizes current knowledge on TBEV–tick interactions, focusing on virus acquisition, dissemination, vector competence, and long-term persistence within tick vectors. TBEV is acquired by ticks during blood feeding on viremic hosts or through co-feeding transmission under experimental conditions. Transovarial transmission has also been reported, as indicated by the detection of infected larvae in nature, although its efficiency appears to be low and variable. Following ingestion, TBEV infects and replicates in the tick midgut before dissemination via the hemolymph to secondary tissues, including the salivary glands and reproductive organs, which are essential for viral persistence and transmission. Vector competence and capacity vary between tick species and are shaped by intrinsic and extrinsic factors. Although transstadial transmission and transovarial transmission contribute to long-term virus maintenance, their efficiency is generally low and variable. In vitro models, including tick cell lines, have provided valuable insights into virus–tick interactions. Nevertheless, important knowledge gaps remain, particularly in understanding early events at the tick–host interface and mechanisms underlying viral dissemination and persistence within ticks.

## 1. Introduction

Tick-borne encephalitis virus (TBEV) is an arthropod-borne flavivirus (arbovirus) belonging to the *Orthoflavivirus* genus within the Flaviviridae family. It is the causative agent of tick-borne encephalitis (TBE), an important neurological disease affecting both humans and animals [1,2,3]. The first description of TBE dates back to 1931, [4] while the virus itself was first isolated in 1939 by Zilber [5]. TBEV is primarily transmitted by hard ticks, which serve both as vectors and reservoirs of the virus. While humans can become infected, they are dead-end hosts, meaning they do not contribute to the natural transmission cycle of TBE [6]. The primary route of human infection is through the bite of an infected tick, but transmission can also occur through the ingestion of raw milk from infected animals [7]. Initially, TBEV was classified into three subtypes: the European subtype, generally associated with a mild form of the disease and a mortality rate of <2% [8]; the Siberian subtype, associated with a higher risk of chronic neurological complications and a mortality rate of around 2% [8]; and the Far Eastern subtype, associated with severe neurological disorders and a mortality rate up to 30% [9]. Recent advancements in genetic screening have resulted in an updated classification system, identifying seven distinct subtypes [10].

TBEV is endemic across Europe and Asia, with over 10,000 human cases reported annually [9]. Between 1974 and 2003, TBE morbidity in Europe increased by 400%, a trend likely driven by improved surveillance, enhanced diagnostics, and environmental changes [2,11,12]. In 2020, a total of 3817 cases of TBE were reported across 24 EU/EEA countries, with the number of cases continuing to increase [13]. In Belgium, TBEV was only recently detected in tick populations, while serological studies in wildlife suggested low-level circulation before the first confirmed autochthonous cases in 2020 [14,15,16].

Ticks are highly sensitive to environmental conditions, and their activity is strongly influenced by temperature and humidity [17]. Warmer climates can extend tick activity periods and may facilitate the emergence of TBEV in new regions [18].

Ticks are obligate hematophagous ectoparasites that require a blood meal at each developmental stage to complete their life cycle. The life cycle of ticks has three development stages: larvae, nymphs, and adults. If eggs are included, four stages are recognized. At each of the active stages, ticks feed once before molting to the next stage or, in the case of adult females, before laying eggs [19]. Hard ticks feed for prolonged periods from days to weeks, depending on their life stage. It takes several years to complete the life cycle, typically between two and six years, depending on environmental conditions [20].

**Figure 1:**
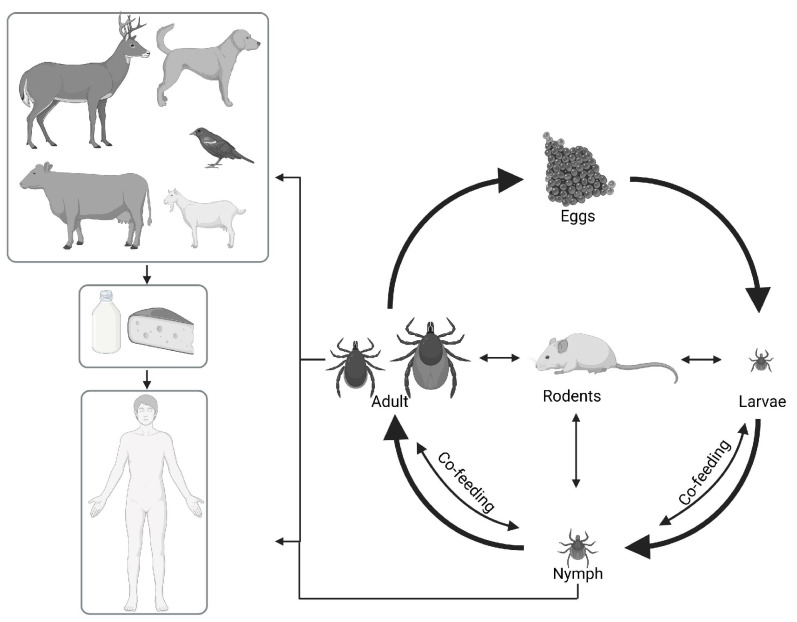
TBEV transmission pathways. Schematic representation of TBEV maintenance in enzootic cycles involving *Ixodes ricinus* ticks and vertebrate hosts. Ticks may acquire TBEV during blood feeding on viremic hosts, through co-feeding transmission, or via transstadial and transovarial transmission. Humans may become infected through tick bites or the consumption of unpasteurized dairy products from infected animals. Created in Biorender. Gabrielle Trozzi. (2026) https://BioRender.com/al40khc (accessed on 12 May 2026).

Ticks feed on a range of hosts, which varies according to the life stage. During the larval and nymphal stages, mainly small hosts such as rodents and birds are used for feeding, while in the adult stage, ticks feed on larger animals (e.g., sheep, cattle, foxes, wild boar, deer). Small rodents are important reservoir hosts because they act as amplifying hosts for TBEV and may contribute to co-feeding transmission, as illustrated in Figure 1 [21].

During feeding, ticks insert their mouthparts into the host’s skin to anchor themselves and then secrete saliva containing immunomodulatory molecules. This saliva not only facilitates feeding but also promotes localized viral replication and non-viremic transmission between co-feeding ticks, a mechanism suggested to contribute to TBEV maintenance. Once attached, ticks increase their body mass through blood engorgement, enabled by their expandable cuticle [19].

While the TBEV replication cycle has been mainly studied in mammalian cell lines, viral replication and dissemination within ticks remain poorly understood [22]. Most existing reviews focus on host- or epidemiology-related aspects rather than on the virus–tick interface. Therefore, the main goal of this review is to summarize current knowledge on TBEV–tick interactions, focusing on virus acquisition, replication, dissemination, vector competence, and long-term persistence within tick vectors. By integrating findings from studies on natural feeding, co-feeding transmission, in vitro tick models, and transmission across developmental stages, this review highlights key mechanisms that enable TBEV to persist in enzootic cycles and identifies critical knowledge gaps in virus–tick biology.

## 2. TBEV Acquisition by the Tick During the Blood Meal (Viremic Hosts vs. Co-Feeding)

Host availability is essential, as ticks need blood meals to complete their life cycle. They detect potential hosts using various sensory mechanisms, including odor detection, vibrations, temperature variations, and changes in light intensity. Hard ticks spend the majority of their life searching for a suitable host. During this period, ticks must conserve water, absorb humidity, and minimize movement to survive. Thermoreceptor cells help ticks locate shelters, while chemosensors assist in identifying hosts [17].

The infection of ticks with TBEV begins with its acquisition by the tick during blood feeding, a process that has been extensively studied. Understanding the process by which ticks acquire TBEV during blood feeding is crucial to explaining its enzootic maintenance and long-term persistence within tick populations.

Initially, it was believed that infection of ticks with TBEV occurred exclusively through blood-feeding of naive ticks on viremic hosts with sufficiently high viral titers [23]. However, experimental studies suggest that co-feeding transmission may contribute substantially to TBEV maintenance in nature [24].

### 2.1. Role of Wild Vertebrate Hosts in the Maintenance and Spread

Wild mammalian hosts, especially rodents, are essential for the maintenance of the viral transmission cycle [9]. Natural hosts, such as field mice (yellow-necked field mice) and bank voles, are key species involved in TBEV maintenance [12]. These rodents are not only frequent blood sources for ticks but also demonstrate prolonged infection persistence, with high TBEV RNA loads detected in blood for up to 1 to 4 months [25], depending on the species, without showing clinical symptoms [26]. TBEV RNA was also detected for up to 4 months in brain tissue [27]. This persistence was long considered the principal source of the virus for tick infection through blood consumption [24].

Numerous other mammalian species are also susceptible to TBEV infection and may develop TBEV-specific antibodies. Most species exhibit only a short and low level of viremia and remain asymptomatic, including domestic ruminants such as sheep and cattle [28,29]. Nevertheless, sporadic case reports of sheep [30], dogs [31], horses [32], macaques [29], etc., with severe symptoms have been reported [12]. The viremic phase in these hosts is transient as the host’s immune system rapidly reduces the viral load. Systemic transmission of TBEV via host viremia appears to be less efficient than non-viremic transmission through co-feeding ticks. Nevertheless, the viremic period can sometimes be sufficient for tick infection during feeding [33]. Birds are also involved in the spread of TBEV despite their low and transient viremia. They contribute to virus dispersal by transporting ticks during migration [34].

### 2.2. Co-Feeding Transmission

**Figure 2:**
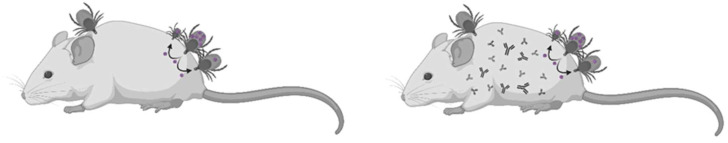
Co-feeding transmission. TBEV co-feeding transmission occurs when a TBEV-infected tick feeds in close proximity to uninfected ticks on the same host. The host serves as a bridge without the need of systemic viremia or even in the presence of antibodies against TBEV (inspired from [24]). Viral particles (TBEV) and antibodies (Y-shaped symbols) are shown schematically. Arrows indicate transmission of the virus from infected to uninfected co-feeding ticks. Created in Biorender. Gabrielle Trozzi. (2026) https://BioRender.com/pfo9jd5 (accessed on 12 May 2026).

It was previously suggested that viremic transmission is not essential for TBEV maintenance in nature [23]. Co-feeding is defined as the transfer of TBEV between infected and uninfected ticks feeding in close proximity on the same host, allowing direct viral transfer at the skin feeding site without requiring systemic viremia (see Figure 2) [24,35]. This mechanism has been demonstrated under controlled experimental conditions and can occur even when the host exhibits low or undetectable viremia or possesses neutralizing antibodies [36]. Rodents may provide suitable conditions for this process, as larvae and nymphs cluster on specific body regions such as the ears, enhancing localized virus exchange [24,35].

Experimental studies on field mice have primarily demonstrated that TBEV can be transmitted between ticks, including on immunized or infected hosts [37,38]. In these settings, infected ticks can release TBEV locally in the host skin shortly after attachment, where it replicates and contributes to transmission. Some field studies support the ecological plausibility of this mechanism. For example, monitoring in endemic areas has shown that bank voles and field mice support simultaneous feeding [38]. It was shown under laboratory conditions that co-feeding on field mice and bank voles can result in a transmission without viremia [39], but this does not directly demonstrate that the transmission occurs under natural conditions.

Experimental knowledge suggests an infiltration of immune cells, including Langerhans cells and other mononuclear cells, that can participate in local amplification and may help in the co-feeding process, as is known for Lyme disease but not fully elucidated for TBEV [35,40]. This mechanism can contribute to virus persistence within tick populations and maintains enzootic circulation even when rodent hosts have acquired immunity.

Controlled experiments in guinea pigs comparing co-feeding transmission with viremia-dependent transmission demonstrated that TBEV is efficiently transferred between ticks, and that this route appeared more efficient under experimental conditions than transmission during systemic viremia. Nevertheless, the physical proximity of feeding ticks on the same host is essential, as it provides the physical platform required for ticks to feed in close proximity [6]. However, this spatial proximity is a parameter enforced by laboratory conditions and remains uncertain in natural conditions.

However, several other hosts—including birds and mammals such as hedgehogs, deer, sheep and goats—can also serve as hosts supporting co-feeding transmission, despite their typically low viremia [41,42]. This suggests that a broad range of vertebrates can support the close spatial feeding required for this process [43].

Several ecological and host-related factors influence the probability of co-feeding transmission. Rodent density can strongly affect the formation of tick clusters, increasing their occurrence up to a threshold after which clustering declines, as shown in yellow-necked field mice [44].

Host traits such as sex and body mass also contribute, with heavier males supporting larger aggregations. Increased deer populations could amplify the tick abundance and the number of ticks feeding on available hosts, such as rodents. Climatic conditions also influence co-feeding opportunities: rapid temperature drops at the end of summer have been associated with increased co-feeding transmission the following year. This phenomenon is due to the synchronization of larval and nymphal emergence in the spring [45].

Overall, co-feeding transmission is considered a potential supplementary mechanism to classical viremic transmission. This process requires temporal overlap of larval and nymphal activity, which is influenced by geographical and climatic conditions [43,46].

### 2.3. In Vitro Systems Used to Infect Ticks with TBEV

Studies using live hosts have provided valuable insights into the mechanisms associated with tick attachment, blood-feeding and TBEV transmission. They nevertheless also have limitations such as ethical concerns, logistical challenges, and host variability. To overcome these issues, artificial membrane feeding and other systems have been developed to provide a controlled and reproducible alternative [24,36].

In addition to artificial membrane feeding, several other in vitro methods have been developed to study TBEV acquisition, persistence and dissemination in ticks. These methods vary in technical complexity and experimental application depending on the research question.

#### 2.3.1. Artificial Membrane Technique

Artificial membrane feeding is the most commonly used in vitro method to infect ticks with TBEV, as it mimics natural blood-feeding while allowing precise experimental control. This system is based on a membrane that simulates host skin, providing a barrier between the tick and the blood, and allowing ticks to attach and feed under controlled laboratory conditions [47].

A major advantage of this technique is the precise control over environmental conditions, such as temperature, humidity, and the composition of the blood meal, ensuring optimal conditions for tick feeding and infection studies [48]. Furthermore, this method allows for the standardization of infection parameters, enabling researchers to regulate virus titers in the blood meal and ensuring consistency in experimental conditions [48,49]. By reducing the reliance on live animal hosts, artificial membrane feeding offers significant ethical advantages, addressing concerns related to animal welfare and experimental reproducibility [50].

Artificial membrane feeding has emerged as a reliable and efficient method for infecting *Ixodes ricinus* and other tick species with TBEV via blood meals, providing valuable insights into vector competence, virus–tick interactions, TBEV transmission and TBEV persistence. Research using these in vitro systems has demonstrated that environmental factors, such as temperature and humidity, play a significant role in influencing feeding success and infection rates [51]. Furthermore, studies have confirmed that natural TBEV vectors can retain the virus during their developmental stages, with the midgut barrier playing a crucial role in the persistence of infection through transstadial transmission [52].

Despite these advantages, artificial membrane feeding has several limitations. The system remains technically complex and lacks full standardization, particularly regarding the membrane composition and device design. In addition, these systems require intensive manual handling and daily monitoring, although partial automation has become available [50]. Furthermore, artificial feeding systems do not fully reproduce the complexity of natural tick–host interactions, including host immune responses and other physiological factors.

By offering a controlled alternative to feeding on a live host, in vitro feeding systems have become essential tools for studying TBEV persistence and transmission. The results obtained with artificially fed ticks will be discussed in later paragraphs.

#### 2.3.2. Capillary Feeding

One such method is capillary feeding, in which ticks ingest a viral suspension from a capillary tube after a brief pre-feeding period on a host. This method mimics natural feeding while allowing precise control over the administered viral dose. The major difficulty of this method is the preservation of the tick’s mouthparts after the capillary feeding [53].

#### 2.3.3. Direct Needle Injection

More direct approaches include rectal and percoxal (hemocoelic) injections. Rectal injection is a technique that introduces the virus directly into the digestive tract via the anus to simulate natural ingestion. The rectal infection method introduces the virus into the gut lumen, and TBEV needs to overcome the intestinal barrier to reach the hemocoel, a limiting step [53]. However, this method is less effective than other invasive techniques for establishing TBEV infection.

Percoxal injection involves injecting the virus into the tick’s hemocoel (body cavity) via the thorax, thereby bypassing the midgut barrier. Studies have shown that bypassing this barrier increases the replication and dissemination of TBEV, confirming that the midgut is an important barrier limiting viral dissemination. While this technique ensures the administration of a controlled viral load, it is technically complex and may negatively affect tick survival [54].

These two methods allow controlled delivery of the virus and facilitate replication and dissemination processes. However both approaches are invasive and can cause substantial physical stress to ticks, often resulting in high mortality rates [54].

#### 2.3.4. Immersion Technique

The immersion technique involves submerging ticks in a virus-containing suspension, allowing infection to occur either through ingestion or passive absorption via the cuticle. This is a simple technique that does not require feeding systems [54,55].

Studies indicate that immersion has low efficiency for TBEV infection and replication, suggesting that passive infection via the cuticle has a negligible role in TBEV acquisition [54,55]. This technique is less used for studying TBEV–tick interaction compared to the other methods.

Despite a growing number of studies focusing on the TBEV acquisition, some aspects of the process of tick-borne encephalitis virus infection are poorly understood. The timing of TBEV entry into the midgut, the crossing of various barriers, its spread in the hemolymph, and dissemination in peripheral organs are still not fully understood. The number of studies remains limited, and variability in experimental approaches reduces the ability to compare results across studies. These factors highlight the need for further research using standardized and biologically relevant models.

## 3. TBEV–Tick Interaction After Blood Feeding

### 3.1. TBEV Replication and Spread Within the Tick

**Figure 3:**
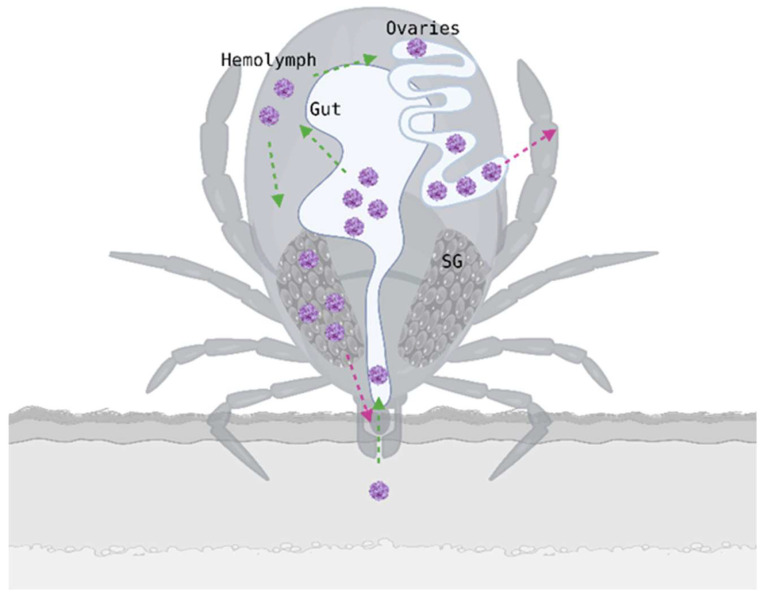
TBEV replication and spread within the tick. Representation of TBEV acquisition during blood feeding. Viral particles are ingested during the blood meal. After ingestion, TBEV invades the midgut and initial replication occurs in midgut epithelial cells, then crosses the midgut barrier and enters the hemolymph, enabling dissemination to secondary tissues such as the ovaries and salivary glands (SG). TBEV is then transmitted to a new host via saliva during feeding or to the next generation via transovarial transmission. Green arrows indicate viral ingestion and dissemination processes, whereas pink arrows indicate transmission routes. Created in Biorender. Gabrielle Trozzi. (2026) https://BioRender.com/0jgmqzj (accessed 12 May 2026).

The replication and spread of TBEV within its vector are essential to understand viral persistence, transmission and vector competence (see Figure 3). However, relatively few studies have examined TBEV replication and dissemination at the level of the whole tick. Most available research examined specific tissues or specific aspects of transmission rather than whole-body viral dynamics. This is probably due to the complexity of working with and infecting live ticks, as well as the biological variability among ticks.

Once ingested during a blood meal, TBEV enters the midgut, where it must overcome several anatomical and physiological barriers before it can infect the tick and eventually be transmitted to a new host. After reaching the midgut lumen, the virus must resist digestive enzymes, antimicrobial factors and the structural barriers formed by the midgut epithelial layer and its underlying basement membrane. As ticks retain undigested blood for extended periods, the virus has sufficient time to initiate infection of the midgut epithelium [56]. TBEV must enter the midgut epithelial cells via receptor-mediated endocytosis or through cell junctions, and this process is mediated by interaction between TBEV and surface molecules on the cells. To date, no specific receptor candidates for TBEV entry have been identified, although studies from other flaviviruses suggest that multiple surface molecules may participate in viral attachment and internalization [22]. Studies on TBEV suggested that the viral envelope (E) glycoprotein plays a central role in host-cell attachment and viral entry. In mammalian cells, the E protein has been proposed to bind to heparan sulfate and other surface molecules that may facilitate viral attachment and internalization. Mutations in the E protein show different effects depending on the cell type. For example, in vivo, decreased pathogenicity and neuroinvasiveness have been observed, whereas in vitro, these mutations increase viral binding to neuronal cells. These findings suggest complex and cell-type-dependent effects on receptor interactions and tropism [57,58]. In tick cells, however, the specific receptors involved in TBEV entry remain unknown. A study on Langat virus infection suggested that plasma membrane proteins on tick cells may participate in viral attachment and internalization, supporting the hypothesis that specific tick-derived entry factors contribute to flavivirus entry [59]. Following entry, viral replication is initiated within epithelial cells.

In addition to these virus–host interactions, the tick gut microbiota is increasingly recognized as an important modulator of pathogen acquisition. This microbiota is composed of both symbiotic and environmental bacteria and contributes to gut homeostasis by modulating gut immunity, maintaining epithelial integrity and producing bacterium-derived components [60,61]. In *I. scapularis*, disruption of gut microbiota has been shown to reduce STAT expression and alter antimicrobial responses [61,62]. These findings suggest that the microbiome may indirectly influence TBEV infection by modulating the gut immune environment and barrier function [63], although direct evidence of microbiome-mediated effects on TBEV remains limited and requires further investigation. Overall, these effects may influence early viral replication and the efficiency of midgut barrier crossing.

A limited number of studies have focused on the innate immune response in ticks against TBEV. It is already known that tick cells respond to TBEV infection by up- and down-regulating specific genes and proteins. Several pathways have been identified as actors, such as the ubiquitin–proteasome pathway, phagocytosis, the piRNA pathway, the unfolded protein response and the complement system. It seems that ticks respond differentially to bacterial versus viral infections [64]. One of the most important antiviral mechanisms is RNA interference (RNAi), a major defense mechanism against arboviruses [65]. This system includes endogenous and exogenous small interfering RNA (siRNA) pathways, components that mediate sequence-specific degradation of viral RNA [62]. The JAK/STAT pathway plays a role in pathogen control through the regulation of antimicrobial peptide expression and maintenance of midgut epithelial integrity [61,62]. In addition, the Toll and immune deficiency (IMD) pathways are also involved in the protection against pathogens within the gut. When they are activated, antimicrobial peptides are generated that can directly kill the pathogens [61]. Together, these immune pathways act at the level of the midgut and likely represent key determinants of TBEV replication and its ability to cross the midgut barrier. Several RNAi components were inhibited by the virus, suggesting their role in the antiviral response [64]. Studies at the whole tick level are lacking to fully understand these immune mechanisms. Blood digestion in ticks occurs intracellularly through heterophagy, a process that enhances viral penetration into epithelial cells [56].

As digestion progresses, TBEV needs to replicate within midgut epithelial cells in order to cross the midgut barrier and enter the hemolymph, enabling systemic spread. The midgut barrier is an epithelial cell layer associated with immune defense that limits viral dissemination [19,56,66,67]. Successful dissemination likely depends on the balance between viral replication efficiency and midgut immune/barrier responses.

Once in the hemolymph, the virus spreads to various organs and tissues, with the highest titers observed in the salivary glands and reproductive tissues [66]. The salivary glands are essential for TBEV transmission, as they serve as replication sites and a long-term reservoir.

Studies have demonstrated that TBEV can cross the midgut barrier and reach the salivary glands, where it undergoes amplification before transmission to a vertebrate host. Viral replication within the salivary glands is necessary to increase the viral load and maximize the amount of virus transmitted to an uninfected host. TBEV can persist in these tissues for up to 120 days post-infection, although persistence duration varies depending on tick species, environmental conditions, and feeding status [68].

Similar to the midgut, the salivary glands possess local immune defenses including the JAK/STAT, IMD and Toll pathways, which are likely involved in controlling viral replication, although their specific roles in this tissue remain incompletely understood. A microbiota is also present in the salivary glands, distinct from that of the midgut. Although it likely comprises diverse microbial species, its composition and diversity remain poorly characterized [61].

While the composition and function of this microbial community remain insufficiently characterized, it is hypothesized that it may influence viral replication or transmission through modulation of local immune responses or interactions with salivary components. However, experimental evidence supporting a direct role of the salivary gland microbiome in TBEV infection is currently lacking [61].

The structure of tick salivary glands enhances viral maintenance. They consist of lobular ducts with three types of acini, each with distinct functions. Type 1 acini are agranular and non-secretory, while type 2 and type 3 are granular and secretory, responsible for saliva production. Male ticks additionally possess a fourth type of acini, illustrating sex-based anatomical differences. During tick feeding, the salivary gland acini expand, and TBEV has been detected within these structures. Persistent infection is localized within these acini and facilitates virus maintenance in nature through transstadial transmission. The transmission of TBEV to a vertebrate host from these glands during blood feeding occurs rapidly, within 15 min after attachment [69,70].

Reproductive organs were identified as one of the tissues with the highest TBEV loads following systemic dissemination at late stages of infection, indicating that these structures play a role in viral persistence within the tick. Laboratory studies provided indications that sexual transmission of TBEV can occur, with infected males transmitting virus to females during copulation via infectious saliva or seminal fluid. Robust experimental evidence for tick–to-tick transmission via copulation remains limited and its ecological significance is uncertain [71,72].

TBEV can persist in unfed *I. ricinus* for at least one year. Ticks collected at the start of the spring season tested positive for TBEV, indicating that the virus can survive overwintering conditions [73]. Such long-term persistence within the tick vector is thought to contribute to the stability of natural TBEV foci and may help maintain viral circulation during periods of low host availability.

Experimental studies have shown that TBEV-infected *I. ricinus* ticks demonstrate altered behaviors. Ticks removed from a host, compared to field-collected ticks, are more active, more aggressive and more resistant to repellents [54].

Overall, TBEV replication and spread within the tick is determined by a complex interplay between viral replication dynamics, tick immune responses, microbiome composition, and tissue-specific barriers in the midgut and salivary glands that remains incompletely understood.

### 3.2. Vector Competence and Capacity for TBEV

Although knowledge about TBEV–tick interactions remains limited, several studies have provided important insights into the vector competence of certain tick species for TBEV, highlighting species-specific differences in acquisition, replication, and transmission.

The term “vector competence” refers to a tick species’ intrinsic ability to acquire, maintain and transmit a pathogen to a susceptible host. This competence is genetically determined and reflects the tick’s capacity to infect, replicate and disseminate within the vector.

In contrast, “vector capacity” encompasses a broader set of factors influencing transmission efficiency, including environmental conditions such as temperature and humidity, as well as ecological and behavioral characteristics such as host preference, host availability, and tick longevity [66,74].

Globally, different tick vector species can coexist within the same geographic regions. Certain species are considered “primary” vectors, as they are adapted to the pathogen and the environment. They are crucial for the maintenance of the pathogen in nature. “Secondary” vectors also exist; these are tick species that can act as vectors if they are competent and present in sufficient numbers. They are not essential for long-term pathogen maintenance [67]. Several tick species have been reported to be involved in TBEV transmission, including well-established primary vectors and additional species with varying levels of experimental support (Table 1). The virus has also been detected in several other tick species, although their role in transmission remains unclear [2,24,29,75].

*Ixodes ricinus* is considered to be the most important vector for TBEV transmission, especially in Europe. TBEV was first isolated from field-collected *I. ricinus* in Czechoslovakia and then from various regions in Europe, supporting its role as the main vector species [56].

A key factor explaining the epidemiological importance of *I. ricinus* is its capacity to transmit TBEV to vertebrate hosts under both laboratory and natural environments. The intimate ecological relation between *I. ricinus* and the vertebrate hosts, especially small mammals, allows co-feeding of different tick stages and contributes to its status as a primary vector [82]. The seasonal overlap of the activity of nymphs and larvae promotes co-feeding on the same host and increases the probability of TBEV transmission [24].

Infection rates were analyzed in *I. ricinus* ticks collected in nature from TBEV endemic and non-endemic regions. The study was conducted over three years at two sampling sites in Germany, where ticks were screened to confirm they were TBEV negative before experimental infection. Ticks were infected by artificial blood feeding and analyzed, and the results revealed temporal and spatial variability in infection rates. Ticks from endemic regions had 2.3-fold higher probability of infection, which may explain the uneven propagation of TBEV. Environmental conditions, particularly temperature and humidity, strongly influence feeding success, for example in tick fitness, a pattern also reported for *Borrelia*-transmitting ticks. Tick age also influences feeding behavior and energy reserves, with older ticks showing lower feeding efficiency [51].

Similar analyses performed from endemic and non-endemic regions showed comparable variability profiles. Infection rates were always higher when ticks were exposed to the TBEV strains that locally circulate, suggesting that local viral adaptation or population-specific tick genetics may influence vector competence. In addition, differences among tick populations may also reflect variation in their associated microbiota, which can modulate susceptibility to infection [83].

Further studies on *I. ricinus* are addressed in the transstadial section (Section 4.1).

Together, experimental transmission studies, ecological characteristics, and repeated field detection provide strong evidence that *I. ricinus* fulfills all criteria of vector competence and represents the dominant TBEV vector in Europe.

*Dermacentor reticulatus* has also been experimentally confirmed as a competent vector of TBEV. This was demonstrated in a comparative study assessing virus acquisition, replication and transmission to laboratory mice (Balb/c) by both *I. ricinus* and *D. reticulatus*. Both species were able to propagate and transmit TBEV, although a lower proportion of *D. reticulatus* individuals became infected despite artificial infection via the coxal plate, and viral titers in positive ticks were also lower compared to *I. ricinus*, indicating species-specific differences in replication efficiency [82].

A co-feeding experiment with *Haemaphysalis inermis,* a tick with a short feeding period, was performed. *H. inermis* nymphs were placed on mice in a chamber together with infected *I. ricinus* to study co-feeding transmission. Results revealed that this species can become infected and provides a suitable environment for TBEV replication and transmission to hosts [82].

For the remaining species listed in Table 1, evidence is limited to either field detection of TBEV or laboratory infection studies, and further work is needed to confirm their role as vectors in natural transmission cycles.

In general, both intrinsic and extrinsic factors determine a tick’s vector competence and capacity. Internally, genetic predisposition and immune responses influence susceptibility to TBEV and its ability to replicate and spread within the tick. Externally, temperature and humidity impact tick survival, feeding behaviors, and pathogen transmission dynamics. Ticks require a humidity level above 85% and an external temperature above 6 to 7 degrees Celsius to remain active and complete their developmental stages, making these parameters key determinants of vector abundance [84].

Climate change further influences these dynamics by accelerating tick development, increasing egg production, and expanding their geographic range. These environmental changes increase tick population density and extend the distribution of competent vectors, which could alter TBEV transmission dynamics. Regions previously unaffected by TBEV may now be at higher risk, highlighting the need for ongoing surveillance and vector control efforts [74,85]. Such alterations can influence host-finding efficiency and feeding success, thus affecting the probability of viral transmission. Despite extensive research on vector competence and TBEV transmission, many aspects remain poorly understood. Therefore, further investigations to elucidate the full mechanisms involved in tick–virus interactions and transmission dynamics are needed.

## 4. Transstadial and Transovarial TBEV Transmission

After establishing infection in the midgut and spreading to the salivary glands and ovaries, TBEV may be transmitted to the next life stage through molting (transstadial transmission) or be passed from females to their offspring (transovarial transmission) [39]. These transmission routes enable long-term virus maintenance within tick populations and make them capable of transmitting the virus to hosts during subsequent feeding [48].

### 4.1. Transstadial Transmission

Transstadial transmission refers to the virus’s ability to persist across molting stages. Because hard ticks only take one blood meal per development stage, the virus must survive molting to remain transmissible during the next feeding [48]. This ability allows ticks to transmit TBEV throughout their lifespan, which can last up to 4–6 years [3,78].

Based on natural observations, transstadial transmission is estimated to occur in approximately 10 to 20% of TBEV-infected ticks that molt to the next stage, depending on tick species and life stage [86]. In contrast, laboratory studies report higher or lower rates depending on how the infection is established, such as infection routes, host involvement, and experimental conditions. These differences likely reflect substantial methodological variation between studies, including the route of infection, the use of artificial inoculation versus natural feeding, differences in viral strains and tick species, and the influence of laboratory conditions on viral dissemination and persistence.

The efficiency of transstadial transmission has also been studied via several laboratory approaches. In a first study, nymphs and larvae of *I. ricinus* and *I. persulcatus* were allowed to feed on TBEV-infected mice and monitored across successive molts. Both species, *I. ricinus* and *I. persulcatus* and their hybrids, were capable of acquiring TBEV and showed evidence of transstadial transmission. For example, with TBEV-Sib infection, 54% of *I. ricinus* nymphs were positive at 9 days post-feeding (dpf) compared to 34% for *I. persulcatus,* but once infection was established, long-term persistence was comparable across strains and tick species. Interestingly, a higher percentage of ticks tested TBEV-positive 49 to 84 days after molting than immediately after molting, indicating long-term viral persistence and replication of TBEV in ticks after molting. The efficiency of transmission and the viral load varied depending on the virus strain used and the tick species. Analysis of adults originating from larvae that had taken an infected blood meal confirmed that TBEV can persist after two successive molts. However, due to the experimental setup, it cannot be excluded that co-feeding transmission during feeding on uninfected hosts may also have contributed to infection persistence. Indeed, nymphs were allowed to feed on uninfected mice in close proximity prior to molting, which may have led to an overestimation of true transstadial transmission efficiency. These findings suggest that transstadial transmission may contribute to long-term TBEV persistence in nature [86].

In another study, nymphs underwent direct inoculation of the virus via the coxal plate, bypassing the midgut barrier. A transstadial transmission rate of 93% was observed from infected nymphs to adults after molting. In contrast, transstadial transmission was much lower when nymphs acquired TBEV via co-feeding. While 61% of nymphs became infected by co-feeding, only 14% of ticks retained the virus after molting to adults. This highlights the complexity of the TBEV maintenance in the tick population and suggests that co-feeding transmission may contribute to virus maintenance under experimental conditions in the tick population, whereas transstadial maintenance is limited by the midgut barrier. Long-term persistence across the different stages is dependent on successful viral dissemination beyond the midgut [68].

Transstadial transmission has also been studied using artificial feeding. Membrane feeding systems allow the investigation of this transmission route without the use of animals, and they produce infection rates similar to those observed in nature [87]. In *I. ricinus* larvae, both membrane feeding and immersion in a virus-containing solution resulted in transstadial transmission after molting to nymphs. Although the TBEV RNA load decreased after molting, nymphs derived from both methods were able to infect mice, demonstrating the successful transstadial transmission [48].

Transstadial transmission has also been shown to occur in another hard tick species, *Dermacentor reticulatus*. Ticks collected from hosts in endemic regions tested positive for TBEV RNA, and this infection persisted after molting, demonstrating that *D. reticulatus* is also capable of maintaining TBEV across developmental stages [78].

### 4.2. Transovarial Transmission

Transovarial (vertical) transmission refers to the virus’s ability to pass from an infected female to her offspring via eggs [48]. Although early hypotheses suggested that this mechanism might contribute substantially to virus maintenance, accumulating evidence indicates that transovarial transmission plays only a minor role in the natural epidemiological transmission cycle of TBEV [37].

Experimental studies have shown that this mode of transmission depends on factors such as viral strain, viral load and tick species, and is generally low, not exceeding 1% of progeny [86]. Not all eggs in an egg batch from a TBEV-infected female are positive; only some eggs are infected [88]. Furthermore, field studies have documented three TBEV-positive larvae collected from small mammals in endemic areas, including one unfed larva, providing evidence that transovarial transmission does occur in nature, although at a very low frequency [89]. In contrast, attempts to experimentally confirm vertical transmission in *I. ricinus* and *R. appendiculatus* yielded negative results: females infected prior to oviposition produced eggs and larvae that tested negative for TBEV, raising questions about the relevance of vertical transmission in these species [90]. A review on this topic concluded that molting and oviposition are generally unfavorable environments for arboviruses, and although transovarial transmission can occur, its efficiency remains low. However, this low transmission is compensated by the fact that larvae from an egg batch feed in proximity on the same host, facilitating infection via co-feeding [39].

More recent studies have confirmed that the efficiency of transovarial transmission varies considerably between *Ixodes* species and that the viral strain also plays a role. Some isolates replicate more efficiently or display strain-specific phenotypic changes depending on the tick species, suggesting vector–virus adaptation. Despite high viral loads in adult females, only a small proportion of eggs or larvae test positive, indicating limited vertical transmission efficiency. In addition, feeding conditions further influence infection outcomes, as virus replication is enhanced during blood meals but may stabilize rapidly afterwards [86,88].

Together, these findings indicate that vertical transmission of TBEV is biologically possible but not consistently efficient. Indeed, this process is restricted by biological barriers, and the infection rates in larvae are also low and variable [90].

All transmission routes, including transovarial transmission despite its low efficiency, contribute to the maintenance and long-term survival of TBEV in nature.

## 5. Tick Cell Lines as a Model for TBEV Research

TBEV has an important public health impact and knowledge on the interaction between TBEV and ticks is important to understand the TBEV transmission cycle and epidemiology. The number of studies addressing this interaction, however, remains very limited. This limitation is due to the difficulty of working with ticks. Maintaining ticks under laboratory conditions is not easy, and experimental work with them is challenging due to their long and complex life cycle. Therefore, in vitro models have been developed as a substitute to study virus–tick interactions.

### 5.1. Diversity and Availability of Tick Cell Lines

In vitro culture systems, particularly continuous cell lines derived from tick and host tissues, play a crucial role in studying tick biology, host–vector–pathogen relationships, and disease control.

Over the years, the number of available cell lines from tick tissues has expanded, with over 40 cell lines derived from 13 ixodid and one argasid tick species. The majority of these cell lines were established by culturing molting larval explants and molting nymphs and have been successfully used to study a wide range of medically and veterinary important pathogens, including TBEV [91].

Tick cell lines offer several advantages over whole-tick experiments, including tightly controlled culture conditions, reduced biological variability, and the ability to support persistent, non-cytopathic TBEV infection, which reflects the natural infection state in ticks. However, they also present limitations: they lack organ-specific tissue architecture, contain heterogeneous, unspecialized cell populations, and do not reproduce physiological signals associated with blood feeding, limiting their ability to model some aspects of virus dissemination and organ tropism. Moreover, tick cell lines do not fully reflect the tissue-specific responses observed in vivo, particularly those associated with the different organs such as the midgut and salivary glands [91,92,93].

### 5.2. Susceptibility of Tick Cells to TBEV and Replication Dynamics

Tick cell lines have contributed to the investigation of the ability of different tick species to acquire, maintain, and transmit pathogens. Initial studies using tick cell lines from five ixodid and one argasid species confirmed that all tested cell lines were susceptible to TBEV infection. However, replication efficiency was highest in Ixodes ricinus for the European strain, suggesting species-specific variations in vector competence [80].

One of the most used tick cell lines in the TBEV research is the IRE/CTVM19, derived from embryonic cells from *I. ricinus.* This cell line originates from homogenized, surface-sterilized tick eggs. This embryonic cell line has been utilized to study viral replication, cellular stress responses, and mechanisms of persistent infection [91]. A study performing mass spectrometry analyses on TBEV-infected IRE/CTMV19 tick cell lines at different times post infection showed that early-stage infected cells (2 dpi) had a different protein expression profile than mid- and late-stage infected cells (5–10 dpi). TBEV infection altered various cellular pathways, including ubiquitin/40S ribosomal protein signaling, ATP synthase function, and histone 2A regulation, processes linked to cellular aging. Additional changes involved alterations in proteasome subunits, heat shock proteins, and mitochondrial ATP synthase, highlighting TBEV-induced modifications in cellular metabolism and stress responses [94].

Another cell line is the IDE8, derived from embryonic *I. scapularis* cells, which has been used to study transcriptomic and proteomic expressions during TBEV infection. Infection of this cell line induced changes in gene and protein expression linked to the innate antiviral defense response of ticks [64]. A second embryonic *I. scapularis* cell line, ISE6, has been used for similar studies, providing additional insights into virus–tick cell interactions and cellular responses to viral infection [91].

Further transcriptomic and proteomic studies on *Ixodes scapularis* and *Ixodes ricinus* cell lines have revealed conserved functional responses to TBEV infection and also species and cell line-specific differences at the gene and protein level. In both systems, genes and proteins linked to nucleic acid processing, metabolism, stress responses, and transport were downregulated, while those associated with immunity and transport were upregulated, illustrating the virus’s ability to manipulate host cellular processes for its replication and dissemination [64].

### 5.3. Other Aspects Studied Using Tick Cell Lines

TBEV persistence during molting, oviposition and diapause are important aspects for understanding TBEV epidemiology but are difficult to study experimentally in live ticks. Tick cell lines have been used as in vitro models to study these processes [95]. Cell lines from different tick species have been shown to support a long-term persistence of TBEV under controlled conditions [91]. In cell lines derived from Ixodes and Dermacentor ticks, TBEV has been shown to persist for at least 120 days. TBEV replicated normally in the tick cell lines during this period but without inducing a cytopathic effect [95,96].

Persistent TBEV infection was associated with increased genetic heterogeneity of the viral population, with the replication and accumulation of viral variants. Persistent infection in tick cell lines can lead to viral diversification and, in some contexts, the emergence of variants with altered virulence characteristics [95]. Neuroinvasiveness in mice was generally conserved despite the loss of cytopathic effect in tick cell cultures. In some cases, cell persistent infection can lead to the emergence of a variant with a reduced neuroinvasiveness, showing that the tick environment can influence the viral phenotype [95,97].

In natural foci, there is a continual transfer of TBEV between hosts and vectors, but under certain conditions, TBEV needs to survive and persist for a long time in ticks [98]. Although viral genetic diversity has been documented, direct evidence linking persistent infection to increased viral heterogeneity in vivo remains limited [95].

TBEV can also adapt to its tick vector through repeated passages in tick cell cultures, leading to the selection of tick-adapted viral mutants. Multiple passages of TBEV in *Hyalomma marginatum* tick cells resulted in specific viral mutations associated with increased fitness in tick hosts and reduced neuroinvasiveness in mice [81].

## 6. Future Directions

Despite an increasing interest in TBEV–tick interactions, several key aspects remain incompletely understood and require further investigation.

A major limitation is the complexity of working with ticks and the experimental constraints associated with laboratory studies. Current techniques are very useful and have significantly helped in the study of tick–host interactions, but they do not always reflect natural ecological conditions. The different experimental infection routes are valuable tools, but they may bypass natural barriers or introduce conditions that are not fully representative of ecological settings. For example, experimental approaches, such as inoculation, bypass biological barriers that would normally influence viral acquisition and dissemination under natural conditions.

At the molecular level, the mechanisms underlying TBEV infection, dissemination and persistence within ticks remain only partially understood. The processes by which TBEV crosses the different barriers, disseminates to the secondary organs and persists within ticks are still partially unexplored. While similar mechanisms have been investigated for other tick-borne pathogens, equivalent data for TBEV remain limited. Similarly, the tick immune system represents a key area of investigation, as its specific role in TBEV infection and persistence remains poorly characterized. A deeper understanding of these interactions is essential to clarify how TBEV is maintained within tick populations.

The relative importance of the different transmission routes, including transstadial, transovarial, and co-feeding transmission, also remains incompletely understood. Although numerous experimental studies have demonstrated the possibility and efficiency of these mechanisms under controlled laboratory conditions, their ecological relevance under natural conditions remains uncertain. Investigating these transmission processes in natural environments remains particularly challenging.

Finally, the TBEV–tick interactions should be considered as a complex system involving the virus, its persistence, tick immune response and environmental conditions. To fully understand this system, integrative studies combining molecular biology, in vitro and in vivo infection models, and ecological validation in natural tick populations will be crucial. The main knowledge gaps and future research priorities related to TBEV–tick interactions are summarized in Table 2.

## 7. Conclusions

This review highlights the complex interactions between the tick-borne encephalitis virus and its tick vectors, which underlie virus acquisition, persistence, and transmission.

TBEV is acquired by ticks during feeding either on viremic hosts or through co-feeding transmission, a mechanism that has been demonstrated experimentally and may enable virus transfer even in the absence of detectable host viremia, although its relevance under natural conditions remains uncertain. Wild rodents serve as key reservoir hosts, while other animals contribute to viral spread and, in the case of birds, the dispersal of infected ticks.

Following acquisition, the virus replicates in the tick midgut before crossing the midgut barrier and disseminating via the hemolymph to secondary organs. TBEV is detected in the salivary glands, which are essential for the transmission to vertebrate hosts. TBEV is also detected in multiple tissues, contributing to persistence across developmental stages through transstadial transmission, while transovarial transmission may occur but appears to play only a minor role in virus maintenance.

Overall, TBEV maintenance in natural foci emerges as a multifactorial process involving efficient acquisition mechanisms, long-term persistence within the tick, and vector–host interactions.

In vitro systems, such as artificial membrane feeding and tick cell lines, have significantly advanced our understanding of TBEV–tick interactions. However, these systems do not fully recapitulate natural transmission processes.

In conclusion, although significant progress has been made, TBEV–tick interactions remain not fully understood. Further research should prioritize integrative approaches combining in vivo and in vitro models to elucidate the mechanisms underlying viral acquisition, dissemination, and persistence within ticks, particularly at the tick–host interface.

## Figures and Tables

**Table 1:** Confirmed and putative TBEV vector species.

Species	Geographical Distribution	TBEV Subtype	Vector Status	Evidence Type	Ref.
*Ixodes ricinus*	Europe	TBEV-Eu	Primary vector	Field detection + experimental	[11,24]
*Ixodes persulcatus*	Russia and parts of Asia	TBEV-Sib, TBEV-Fe	Primary vector	Field detection + experimental	[24,76]
*Haemaphysalis concinna*	Europe and parts of Asia	TBEV-Eu	Secondary vector	Field detection + experimental	[21,76,77]
*Dermacentor* *reticulatus*	Europe and Western Asia	TBEV-Eu	Secondary vector	Field detection + experimental	[78,79]
*Haemaphysalis* *inermis*	Europe	TBEV-Eu	Putative vector	Limited experimental	[20,80]
*Ixodes arboricola*	Central Europe	TBEV-Eu	Putative vector	Field detection	[76]
*Haemaphysalis punctata*	Central Europe	TBEV-Eu	Putative vector	Rare reports	[3,29]
*Dermacentor marginatus*	Europe	TBEV-Eu	Putative vector	Laboratory infection	[81]

Abbreviations: Eu: Europe, Sib: Siberian, Fe: Far-Eastern.

**Table 2:** Overview of current knowledge, limitations, and future research directions regarding TBEV–tick interactions.

Biological Process	Current Knowledge	Limitations	Future Research
Co-feeding transmission	Experimentally demonstrated transmission route with uncertain ecological relevance	Evidence only in laboratory conditions	Studies of co-feeding transmission under ecological conditions
Viral acquisition	TBEV can enter and infect the midgut epithelial cells via endocytosis or cell junctions	No information about cell-specific receptors	Elucidate the molecular mechanism and receptors used for viral acquisition
Tick microbiota	Important modulator of pathogen acquisition	No characterization of microbiome-mediated effect on TBEV	Investigation of how microbiome composition influences TBEV susceptibility
Viral dissemination within ticks	TBEV disseminates from the midgut to secondary tissues	Mechanisms and kinetics of viral dissemination remain poorly understood	Investigation of viral dissemination kinetics in tick tissues using in vivo and in vitro models
Tick immune response	Tick cells respond to virus infection by different immune pathways (RNAi, JAK/STAT, Toll)	Tick-specific immune pathway to TBEV infection is poorly understood	Identify the specific immune pathways involved in TBEV infection control in vector species
Salivary gland microbiome	Role in the infection and transmission process	Composition and role are not fully characterized	Identification of the composition and function of the salivary microbiome and its role in TBEV infection
Viral persistence	Laboratory proof of virus persistence during long periods within ticks	No information on long-term persistence under natural conditions	Investigate long-term viral persistence in terms of ecological relevance
Transstadial and transovarial transmission	Role in the TBEV maintenance within tick populations	Unclear information about the ecological relevance under natural conditions	Field studies to quantify the occurrence of these transmission routes

## Data Availability

No new data were created or analyzed in this study. Data sharing is not applicable to this article.
